# Factors Involved in the Immunological Protection against Rabies Virus in Dogs in Spain

**DOI:** 10.3390/vaccines12030293

**Published:** 2024-03-11

**Authors:** María Dolores Manzano, Javier Cereza, Jesús García, Luis Javier Yus, Juan José Badiola, Juan Emilio Echevarria, Marta Monzón

**Affiliations:** 1 Research Centre for Encephalopathies and Transmissible Emerging Diseases, Institute for Health Research Aragón (IIS), University of Zaragoza, 50013 Zaragoza, Spain; mmanzano258@alumno.uned.es (M.D.M.); badiola@unizar.es (J.J.B.); 2 Programa de Doctorado en Ciencias Biomédicas y Salud Pública UNED-IMIENS, Universidad Nacional de Educación a Distancia (UNED), 28015 Madrid, Spain; 3 Centro Técnico Veterinario (Diputación de Zaragoza), 22002 Zaragoza, Spain; recagrar@dpz.es; 4 Colegio Oficial de Veterinarios de Zaragoza, 50010 Zaragoza, Spain; presidencia@veterinarioszaragoza.org (J.G.); gerencia@veterinarioszaragoza.org (L.J.Y.); 5 Centro Nacional de Microbiología, Instituto de Salud Carlos III, Centro de Investigación Biomédica en Red de Epidemiología y Salud Pública (CIBERESP), 28029 Madrid, Spain; jeecheva@isciii.es

**Keywords:** factors affecting seroprevalence, immune protection against rabies virus in dogs, One Health, rabies vaccine, serum antibodies

## Abstract

Rabies, a viral disease spread by infected animal bites that causes encephalitis in humans and other mammals, is a neglected infectious disease present on all continents except Antarctica. Spain has been free of terrestrial rabies since 1978. However, due to its geographical situation, it represents a bridge for imported cases from an endemic continent such as Africa to Europe. Rabies vaccination in dogs is an essential preventive tool against this zoonosis. The aim of this study was to determine the state of the immune response against rabies virus in dogs in Spain and to demonstrate whether several factors that have been previously related to the influence of the seroprevalence of this species are involved here. The seroconversion level of this zoonotic virus was assessed in a total of 1060 animals. Indirect ELISA was used to obtain data for statistical analysis to evaluate the studied variables. Working under the concept of One Health, this study provides relevant information to be taken into consideration not only to prevent re-emergence in countries free of this disease but also for prevention and control in endemic countries.

## 1. Introduction

Rabies is one of the neglected infectious diseases prioritized by the World Health Organization. It is caused by *Lyssavirus* (Family *Rhabdoviridae*), usually transmitted by the saliva of dogs and bats through sick animal bites. It affects the central nervous system of mammals, involving nervous symptoms and behavior changes in both humans and animals, the associated encephalitis being close to 100% lethal.

Today, the rabies vaccine described by Louis Pasteur in 1885 is still considered one of the most important tools for rabies prevention [1]. However, despite more than 100 years since its discovery, approximately 60,000 people die from rabies each year [2,3].

Among the main risks of the presence of rabies in an area are the population of free-roaming dogs as well as the decrease in so-called herd immunity [4]. A study in Zimbabwe revealed that the odds of a dog causing rabies are greatest among unvaccinated individuals [5], so the canine population is the main target of rabies vaccination. The World Health Organization (WHO 2021) considers that vaccination of this species is the most cost-effective strategy for the prevention of rabies since it would not only reduce deaths due to this disease but also have a great impact on reducing the costs of postexposure rabies application and control of outbreaks.

The purpose of this vaccination is to provide immunity against exposure to the virus, not only protecting animals against the disease but also generating adequate vaccination coverage to achieve group immunity. Different authors agree that to sustain such herd immunity, a coverage of the population greater than 70% is required [6,7], and in fact, the WHO recommends that in endemic countries, there should be a canine coverage of at least 70%.

Different countries have achieved canine rabies elimination through mass vaccination [8]. However, in some of them, coverage has not been subsequently maintained, generating a risk of reintroduction of the disease. It is worth noting that rabies, not only in domesticated but also in wild animals, is harmful to dogs. In 2017, it was estimated that worldwide rabies vaccination coverage in dogs was less than 20%, with the exception of the American continent, which is an endemic region [9]. In developing countries, which are endemic regions, the cost of vaccination is not affordable for the population, and access is difficult, particularly in rural areas, which are the regions with the highest incidence [10]. Consequently, reducing human death and investment in rabies treatment is necessary to improve canine vaccination coverage. The most effective strategy involves providing financial and logistical resources and applying the “One Health” concept, with medical and veterinary authorities working together [11].

Regarding vaccination schedules in Spain, currently, immunization is compulsory in most of the Autonomous Communities, except in Galicia (Catalonia is currently making it compulsory). Based on this mandatory vaccination, reliable conclusions about the cause of the lack of protective titers are expected to be drawn from this study. However, there is no strategy for determining the state of immune protection against the virus in vaccinated dogs. This fact makes it difficult to estimate the risk of spreading the disease in the event of an imported case of rabies and contact with dogs or other susceptible animals. According to studies by the Affinity Foundation in 2018, 138,000 dogs and cats (75.86% and 24.43%, respectively [12]) were picked up or found on the street in Spain. In this study, we focus on the problems of canine species, although the importance of feline species in public health is even more understudied.

Only one study published in 1997 on vaccinated dogs in two Spanish areas was found in the literature, with the aim of determining the seroprevalence of the rabies virus. According to our results, of the 156 animals studied, only 58.3% presented optimal levels of protection against rabies [13]. The data collected in 2017 by the Official Association of Veterinarians of Aragón show that in this Autonomous Community, the number of animals that had been vaccinated against this disease decreased by more than 40% over the previous year (MSD Animal Health, 24 September 2018).

The immunogenicity of rabies vaccination can be evaluated by measuring the presence of rabies virus-neutralizing antibodies in the serum. Protection is not assured by seroconversion. The use of “protection” in the manuscript refers to the evidence of a high probability of survival from a rabies challenge associated with seroconversion (level ≥ 0.5 IU/mL) based on the summary of challenge studies cited in the document. According to WHO recommendations, 0.5 international units (IU)/mL detected by ELISA (which, using rabies glycoprotein, has been shown to have good correlation to neutralizing antibody) indicates an adequate immune response after vaccination against rabies, and as a consequence, it is considered a reasonable level of seroconversion in animals [14].

The failure of vaccine immunity is related to different factors, such as a lack of knowledge and training of the personnel administering the vaccine in the use and handling of the vaccine, inadequate maintenance of the cold chain or lack of adherence to the general recommendations of the laboratory [15]. In addition, the period between vaccination and sampling can logically influence the presence or absence of antibodies as well as titer levels [16]. There are patients receiving rabies vaccines whose time after vaccination when the individual will be protected from the disease is 1 to 3 years. On the other hand, some authors have shown that a single dose of rabies vaccine, despite the generation of antibodies, does not achieve satisfactory protection, requiring a booster vaccine to achieve immunity [17]. Thus, some experts suggest a specific schedule for dogs with no vaccination history by administering two other booster doses after the first dose, one at 30 days and one at 180 days, to achieve annual protection [18]. Another later study revealed that in dogs that had already achieved a protective titer after vaccination, their immunity decreased to 58% by day 90 and 35% by day 360, suggesting that booster vaccination should be administered 3 months after the first vaccination to ensure that antibody levels persist for at least one year [19].

Referring to individual factors of the animal itself that are mostly involved in vaccination efficacy, such as a lack of capacity for generation or maintenance of the level of protection [19], some studies propose immune insufficiency or an impaired immune response due to thymus degeneration [20]. Although it is not entirely clear yet, some researchers suggest that the primary response to vaccination may be impaired in older dogs [21].

Among the well-known factors correlated with the immune response are breed and body size. Some studies have reported that larger purebred dogs (medium-very large) are less likely to achieve an optimal antibody level than smaller purebred dogs (small-very small), suggesting that a booster dose should first be applied after primary vaccination [22]. Furthermore, concerning the quality of animal nutrition, better postvaccination protection in well-nourished animals [23] and nutritional deficiencies interfering with the immune response to some vaccines, especially in puppies, have been shown in previous studies [24]. As another immunological factor, stress associated with different situations [25] represents an important factor potentially responsible for a lack of immunity. In some housing conditions, such as shelters or kennels, due to isolation and social restriction [26], confinement prior to shipment [27] or transport itself, in the case of animals coming from different countries [28], temporary or chronic stress is expected in dogs. Moreover, in police, military, firefighter dogs, etc., this effect could also be enhanced by other factors, such as exposure to different diseases and unknown environments, contact with wild or feral animals or people acting as possible vectors of different diseases, or exposure to contaminated areas and material by using their sense of smell to detect drugs, blood, etc. [29].

In addition, since individual resistance and susceptibility to infection and the response to vaccination may also be influenced by hormones [30], although some studies in dogs have shown no sex difference [31], it has generally been shown that the immune response tends to be more vigorous in females than in males [32].

After an exhaustive bibliographic review of such scarce studies published regarding the situation in the national territory of Spain, only one, to our knowledge, it has been revealed as indispensable to know the state of the immune response against the rabies virus in dogs in Spain. This study also aimed to determine whether immunological factors or features of vaccine administration or schedules that have been previously considered by other authors to influence immunity affect the demonstrated seroprevalence.

## 2. Materials and Method

**Animal groups and data collection.** This serological study of rabies virus antibodies was approved by the Ethical Advisory Committee for Animal Experimentation of the University of Zaragoza (PI45/16).

A total of 1060 serum samples from dogs were included in the study and classified into 5 main groups depending on the population to which they belonged:

- Attending veterinary clinics: A total of 383 serum samples from dogs in the province of Zaragoza were collected when the dogs visited a veterinary clinician.
  In all cases, the owners signed the corresponding consent to participate in the study.
- Working dogs: Sera were collected from 320 *Guardia Civil* animals (trained for different purposes: security, search and rescue of people, explosives, poisoned bait or drug detection, paper money, accidents, etc.) located in the facilities of the *Escuela de Adiestramiento de Perros* (CADEPE-SECIR) in *El Pardo* (Madrid) or from those on duty in different Spanish regions.
  The SECIR Director Board and handlers responsible for dogs were informed of the purpose of the study and agreed to participate. All the dogs were located in kennels.
- Housed in shelters: these 269 samples mostly came from stray dogs (constituting, as indicated above, the main population at potential risk in the context of rabies cases).
  Sampling was carried out in the following facilities from the Region of *Aragón*: the Animal Protection Center of the *Diputación de Zaragoza*, the Animal Shelter of Zaragoza, the Shelter and Animal Protection *Amigo mío* from *Teruel* and the Animal Protection Center of the *Diputación de Huesca*. In addition, samples were collected from shelters in Spanish regions other than Aragón, such as País Vasco, Galicia, Madrid, Murcia, and Andalucía. All of them were provided by the Spanish Association of Municipal Veterinarians.
  Dogs from this group were housed in kennels.
- Animal nursery: Sera were obtained from 60 dogs located in the same nursery, but information from this group was scarce. In fact, it consisted of only the Jack Russell Terrier and Dachshund (Dachshund) breeds, which were hosted in kennels.
- Housed in research animal facilities: A total of 28 serum samples from experimental animals located in kennels at the facilities of the University of Zaragoza were included. All of them were from dogs of the Beagle breed and were obtained from a breeding facility in France.

All the available information was collected from owners, handlers or persons in charge of shelters and, whenever possible, from passports or health cards. An attempt was made to collect data regarding size, age and sterilization; breed (according to the criteria established by the *Fédération Cynologique Internationale*, FCI, and the *Real Sociedad Canina de España*, RSCE) to classify potential dangerousness (including, as classified in Spain, Pit Bull Terrier, Staffordshire Terrier, American Staffordshire Terrier, Rottweiler, Dogo Argentino, Fila Brasileiro, Tosa Inu and Akita Inu); geographical origin (referring to the country of birth); environment and habitat where they lived; behavior (if social); state of health (in case illness, the type of disease was indicated); date of the last antirabies vaccination prior to sampling (type of vaccine, commercial brand and lot number); and number of doses applied.

**Screening for inclusion.** Owned, working and experimental dogs that were eligible for inclusion in the present study had to have received rabies revaccination or one dose applied at least one month before the day of blood collection. All commercial vaccine brands were accepted, regardless of the expected duration of immunity (they are all routinely used in veterinary clinics because they are approved by the World Organization for Animal Health (WHO Terrestrial Manual 2021). For all dogs from the animal nursery and most of those housed in shelters, the vaccination status was completely unknown.

**Sample collection.** Five milliliters of blood were extracted by cephalic vein puncture and refrigerated in tubes without anticoagulant.

All of the samples were processed at the Research Centre for Encephalopathies and Transmissible Emerging Diseases at the University of Zaragoza immediately upon receipt. All the samples were centrifuged (3100 rpm; Kubota 5930, Japan) for 10 min and subsequently stored frozen (−20 °C) until antibody titration.

**Assessment of serum antibody levels.** The PLATELIA RABIES II Kit, which is based on an indirect immunoenzyme technique capable of recognizing specific antibodies against rabies virus glycoprotein [33,34] was used for this study.

**Statistical analysis.** All the data collected were subjected to statistical analysis. The protocol was applied following the manufacturer’s recommendations for diluted samples (1/10). The results were classified as a high level of seroconversion (>4 IU/mL), recommended (0.5 to 4 IU/mL), insufficient (0.125 to 0.5 IU/mL) or undetectable (<0.125 IU/mL).

The following database included the categories within each variable, as shown in Table 1: population (as the main group), behavior, dangerousness, sterilization, habitat, size, last vaccine, origin, and body condition. R* software (R Core Team, Vienna, Austria, 2022) was used for all analyses.

To study the relationship of the main variable, the seroconversion level (i.e., >, < or = 0.5 IU/mL), with the rest of the variables studied, hypothesis contrasts were used. *p* values (*p* values overall) are indicated for each case.

For quantitative variables (age and number of vaccines administered), goodness-of-fit tests were used to determine whether the variable was likely to come from a specified distribution. Nonparametric tests were applied, providing indicators of central (median) and noncentral (quartiles 1 and 3) tendencies.

To illustrate the relationship between quantitative variables, box-violin graphs were used. Nonparametric contrast measures are shown in the graph. The Mann-Whitney U test was used to compare two groups, protected vs. unprotected, and the Kruskal-Wallis test was used for comparisons among 4 groups (antibody TITRES, IU/mL): <0.125, <0.5, ≥0.5 and >4). In the latter case, statistically significant two-by-two comparisons were added by applying Holm’s correction for multiple comparisons.

For qualitative variables, chi-square tests were applied, and the results are presented as frequencies and percentages in each category. In addition, the level of protection of a category with respect to the reference category (Ref) was compared using the odds ratio (OR), providing its 95% confidence interval and presenting the *p* value associated with this OR.

The use of “protection” in this study refers to evidence of a high probability of survival associated with a level ≥ 0.5 IU/mL based on the studies cited throughout the document.

## 3. Results

According to the total number of serum samples analyzed in this study, the group with the highest number of individuals was the group corresponding to veterinarian clinics, represented by 383 animals (36.13%), followed by working dogs, with 320 (30.19%), and those from shelters, with 269 (25.38%). The number of those corresponding to animal nurseries decreased to 60 (5.66%) and to 28 (2.64%) for those involved in research.

A statistical analysis revealed significant differences (*p* < 0.001; Table 1) between the different groups. Dogs in shelters were up to 20 times more likely to be unprotected than dogs attending clinics with their owners (OR = 0.05) or almost 16 times more likely to be unprotected than animals trained for security or rescue work (OR = 0.06). In animals from breeding or experimental farms, the probability of not presenting protective antibodies against the virus also decreased, although to a lesser extent (OR = 0.36 and 0.27, respectively).

Considering the behavior of the animals, the probability of not being protected increased more than twofold for the more sociable dogs (OR = 2.33, *p* < 0.001). Even more so when considering animals of dangerous breeds (OR = 2.74, *p* = 0.003).

Among the individuals included, 58.29% were males, 41.71% were females, and 17.90% had been sterilized. This procedure was shown to be a protective factor (OR = 0.15, *p* < 0.001), decreasing the probability of nonprotection by almost seven times.

With respect to habitat, it was shown that animals living in cages (kennels) had a 6-fold higher probability of being protected than those living in a garden house or on a farm (OR = 6.01, *p* < 0.001).

When comparing the variables studied in all animals according to size, a 4-fold greater risk of not presenting protective antibodies against the virus was detected in medium-sized animals than in small animals (OR = 4.11, *p* < 0.001).

The same factor, four, was observed when comparing the type of last vaccine administered. In this case, the polyvalent vaccines (EURICAN MHP-LR, CANIGEN MHA2PLR, VERSICAN DHPPi/L3R, NOBIVAC RL or EURICAN DAPPi-LR) were shown to exert significantly stronger protection versus the monovalent vaccines (NOBIVAC, RABISYVA VP-13, VERSIGUARD RABIA, EURICAN R, RABIGEN L, ETADEX or RABISYVA VP-13; OR = 0.24, *p* < 0.05).

The optimal condition of the animal also acted as a protective factor, increasing to almost double the probability of presenting with serum antibodies (OR = 1.77, *p* < 0.05).

For the variable of origin, no significant differences were observed. However, concerning this last category, it should be mentioned that the number of dogs whose origin was Eastern Europe accounted for only 1.07% of the animals, so it was not possible to calculate the OR.

**Figure 1:**
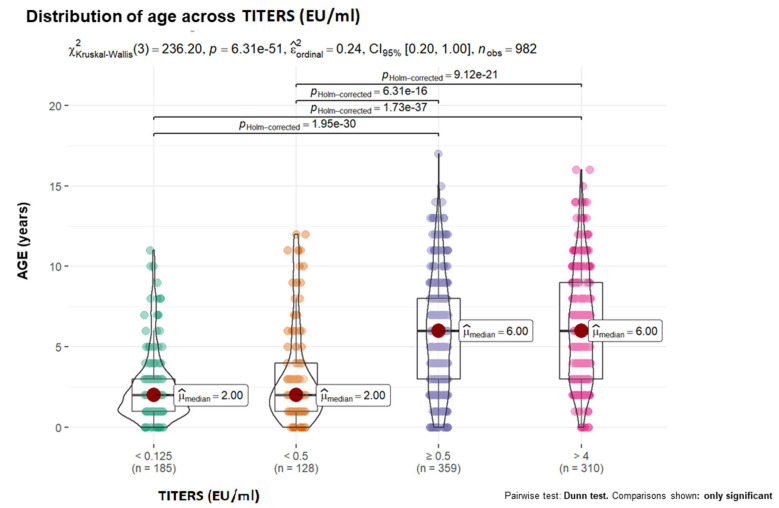
Distribution of individuals by age and seroconversion level.

**Figure 2:**
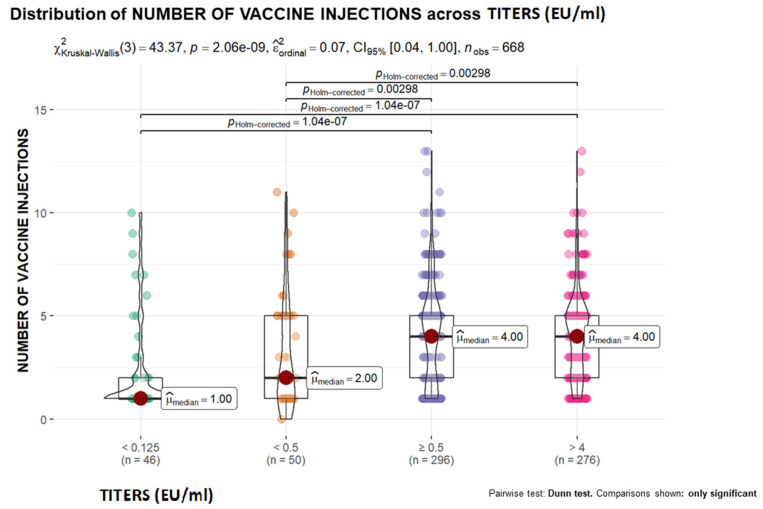
Distribution of individuals according to the number of vaccines administered and seroconversion level.

With respect to age, greater protection was observed as age increased (Figure 1). A similar tendency was demonstrated for the other quantitative variables, as the probability of showing protection increased as the number of vaccine doses administered increased (Figure 2).

Overall, in the present study, 66.8% of the animals analyzed had adequate protection against the virus. Moreover, when four categories of protection were established (<0.125, <0.5, ≥0.5 and >4) instead of two categories (protected, ≥0.5 and not protected, <0.5), 30% of the animals had a titer higher than 4 IU/mL, and 20% had a titer less than 0.125 IU/mL. If the protection variable was analyzed with these four categories (Table 2), *p*. trend < 0.001 was observed between the categories of all the variables except for the origin variable, which, again, did not reach significance (>0.05).

Most notably, in the shelter group (*p*-trend = 0.000), the highest percentage of animals (63.16%, 132 animals) had the lowest titer values (<0.125), and only 16 (5.03%) had the highest titer values (>4). However, in the groups of dogs from veterinarian clinics and working animals, the proportions were inverted, with the lowest titers presented by 27 (12.92%) and 25 (11.96%) animals, respectively, in comparison with the highest titers presented by 182 (57.23%) and 116 (36.48%) dogs, respectively. The same trend (*p*-trend = 0.000) was found when analyzing the sterilization category; in the sterilized animals, only 8 presented the lowest serum titers, while the majority of them, 99 (31.83%), presented the highest titer values (>4). However, in the case of unsterilized animals, the percentages were very similar for both extremes of titers, 179 (95.72%) and 212 (68.17%).

## 4. Discussion

Some studies comparing the efficacy of reducing rabies titers in dogs with different living environments by influencing their health and immune status have been published. However, to our knowledge, neither the specific populations nor the determining factors included here have been assessed before. In Spain, only one study on this seroprevalence has focused on a few variables and cases [13]. The results of the present study provide relevant information related to different factors to be taken into consideration not only to avoid the re-emergence of rabies in Spain and in rabies-free countries but also for prevention and control in endemic countries. Spain has been free of terrestrial rabies since 1978. However, due to its geographical situation, it represents a bridge for imported cases from an endemic continent such as Africa to Europe. Specifically, more than 100 imported cases of rabies have been reported after 1978 from the Spanish territories of Ceuta and Melilla (coming from North Africa [35]). There is no evidence of importation of infected dogs from Ceuta and Melilla into mainland Spanish territory or other European countries. Meanwhile, according to epidemiological data from 2017, 234 cases of canine rabies, 15 cases of human rabies and 65,000 cases of human exposure have been reported in Morocco [36]. In this case, importation of infected dogs from Morocco into Europe has been frequently reported [37].

One of the essential novelties of the present study is the inclusion of working dogs, a population that has been very scarcely assessed in serological studies. Here, 83.4% presented adequate rabies protection, whereas a study conducted in rabies-vaccinated police dogs in China reported that 67.91% were protected, and another study on military dogs in Korea reported that more than 90% were protected [38]. These findings indicate a very low risk of transmission to human and animal populations in contact with these working animals in different national or international scenarios [39]. Nevertheless, as they are constantly exposed to different environments, are in contact with other working canine units [40] or even wild animals and are trained to bite in certain scenarios related to their profession [41], they become more vulnerable to becoming infected and transmitting infections.

Nursery animals represented another novel group of dogs assessed in the present study. Despite the high demand for purebred dogs that currently exists and the fact that some of these facilities do not comply with corresponding legislation [42] and are associated with animal health problems and lack of animal welfare [43], there are very few studies on this population of dogs. On some occasions, animals are housed in unprotected cages in open fields and are exposed to contact and bites from wildlife (which can be infected with rabies) [44,45]. Moreover, transmission of potential infection by direct contact during crossbreeding should not be excluded, even though rabies transmission through semen has not been demonstrated [46]. The risk factors that constitute the failure to achieve an immune response for housing, transport, sale, purchase or attendance at shows in these dogs should be considered [47].

Dogs housed in research facilities for experimental studies, another understudied population, were confirmed to exhibit greater rabies protection than stray dogs. This observation confirms that adequate health, feeding and handling are relevant aspects for improving the efficacy of vaccines.

Additionally, an aspect to take into account is that not all countries require rabies titration for entry into their territory, as the vaccination booklet is sufficient. The falsification of rabies vaccination documents is also a recognized risk for imported pets. Based on the results obtained in this work, where both animals from the animal nursery and experimental facilities did not reach 100% protection, it would be advisable to act in the same way [48]. Titration of antibodies before the sale of dogs would be desirable.

Overall, this work demonstrates that the place of residence influences whether dogs obtain appropriate immunization against rabies. Living in kennels represents a factor that clearly decreases the protective role of vaccination in relation to the other housing groups. Stress likely alters immune processes and functions [49,50]. Confinement in cages (kennels) in shelters, kennels or research centers, representing unfavorable and noisy environments, exerts an evident influence on the immune system [51], increasing the susceptibility of animals to infection [52].

Another factor that influences the serological status according to the results provided here is animal size. Medium-sized animals exhibit less protection against the virus in comparison with small animals (a four-time greater risk, specifically). Similar observations have been described in a study in Sweden [22], suggesting that this is due to genetic factors [53]. Moreover, others considered the possibility of a sequestration of antigen through the fat that is present at the subcutaneous level at the sites where the vaccine is most frequently applied [54,55]. Regardless, the special relevance of this factor is that medium-sized dogs are responsible for the greatest number of aggressions [56]. In addition, guide dogs are within the group of medium-sized canids [57], being very vulnerable to attacks and bites from other dogs and consequently putting not only the animal at risk but also its owner [58]. However, the special relevance of paying attention to this influential factor is that in Spain, most abandoned dogs that enter shelters are of this medium size [59].

As also evidenced in this work, a worrying element to consider is temperament. The probability of being unprotected increases more than twofold for the most sociable dogs. The most sociable group includes animals under one and a half years of age [60] that interact more frequently with children, adults and other dogs [61]. In a study in Thailand, 14% of dogs with rabies were less than 3 months old, and 42% were 6 months old [62], while in Mexico, the average age of rabid dogs was one year old, and 98% were owned [63]. Moreover, owner–dog bonds are becoming increasingly closer (especially in more developed countries [64], as they are being considered family members) and increasingly more sociable [65]. In fact, there is an increasing number of dog facilities as part of pet marketing that are dog friendly, which must be regulated to prevent and reduce potential risks and the transmission of infectious diseases [66].

Specifically, when dealing with aggressiveness, a greater probability of not achieving adequate protection was observed. This finding could be a cause for alarm since, as evidenced in a 10-year period report in Spain about fatal dog attacks (reporting 1.6 cases/year), aggressor breeds were mostly identified as dangerous breeds: Pitbull and its crosses, Rottweilers, Akita Inu, Doberman and German Shepherd [67]. Moreover, other reported findings indicate a high number of bites by dangerous breed dogs [68,69,70].

Another conclusion drawn from the results of this study is that unsterilized males are more likely to be unprotected. A recent study in India supports these conclusions, showing that neutered dogs are approximately four times more likely to present antibody titers than unsterilized dogs [71]. Thus, it is indisputable that an animal’s reproductive status and hormones, mainly testosterone, influence the immune response [72]. This observation, combined with the fact that unsterilized dogs, especially males, have a greater risk of biting [73] and that sterilization is one of the most effective measures for reducing aggressive behavior [74], could have very important implications for sterilization campaigns. Surgical sterilization is strongly supported, as it could be used not only to reduce male wandering and fights [75] but also as an additional method of rabies control [76].

With respect to age, older animals exhibited greater antibody titer responses than younger animals, suggesting that age is a protective factor. Some studies have shown that young dogs have a lower probability of achieving adequate protection [77,78]. Most likely, the real cause is, as previously suggested, the increased number of doses administered to the animal, which consequently increases its protection [72]. In contrast, in endemic areas, rabies cases have been reported in puppies under three months of age, even in some who are less than one month old, so the WHO recommends vaccination of this age group in risk areas [61]. In addition to the lack of maturity of the immune system, maternal protection is considered the main cause of the lack of vaccine protection in puppies [79], as no protective antibodies from vaccinated dams have been identified [80]. This finding reinforces the importance of applying a booster dose at the time of first vaccination before the annual rabies booster [81]. Moreover, based on the immunological state of individuals according to age, other publications have described how dogs younger than 6 months are unprotected [62], those between 6 months and 5 years present the greatest protection, and those over 9 years show a decrease or a tendency toward decreased acquired protection [78].

Finally, contrary to some articles indicating that monovalent vaccines generate higher titers than polyvalent vaccines [81,82,83], the analysis presented in this study revealed that a polyvalent vaccine as the last vaccine administered exerted a higher protection than a monovalent vaccine. This could suggest this type of vaccine for the booster dose.

In summary, variables that had never before been included in a study of this type were analyzed here. From the point of view of the competent authorities, this study will help to better understand the different factors that influence the achievement of adequate rabies immunity in dogs and to take these factors into account at the time of the establishment of vaccination programs. Notably, there was a potential decrease in the economic loss caused by bites and other injuries, which in 2020 had a cost close to USD 854 million in the USA [84].

Finally, under the concept of One Health [85,86], rabies is prevented by raising awareness and education. Considering the conclusions drawn in this study, attention should be given to the impact of animal welfare, ensuring the optimal conditions of the facilities, in order to achieve good immunization, as well as ensuring more rigorous control at EU border posts [87], emphasizing the importance not only of rabies vaccination but also of titration, especially if the animals are transported and/or carry out their work nationally or internationally [88].

## Figures and Tables

**Table 1:** Relationship between the main variables studied and seroconversion level in IU/mL by hypothesis testing (Ref = reference category).

Variable	% Animals/Total Number N = 1060	% Protected Animals ≥ 0.5 N = 708	% Unprotected Animals (<0.5) N = 352	*p*-*Overall*
				*Odds Ratio*, OR (95% Confidence Interval)
**Population (Main group)**				<0.001
Shelters	269 (25.38%)	64 (9.04%)	205 (58.24%)	Ref
Vet clinics	383 (36.13%)	334 (47.18%)	49 (13.92%)	0.05 (0.03; 0.07)
Animal nursery	60 (5.66%)	28 (3.95%)	32 (9.09%)	0.36 (0.20; 0.64)
Research facilities	28 (2.64%)	15 (2.12%)	13 (3.69%)	0.27 (0.12; 0.61)
Working	320 (30.19%)	267 (37.71%)	53 (15.06%)	0.06 (0.04; 0.09)
**Behavior**				<0.001
Not sociable	112 (11.37%)	93 (13.73%)	20 (6.35%)	Ref
Sociable	873 (88.63%)	578 (86.27%)	295 (93.65%)	2.33 (1.44; 3.97)
**Dangerousness**				0.003
No	947 (96.14%)	653 (97.46%)	294 (93.33%)	Ref
Yes	38 (3.86%)	17 (2.54%)	21 (6.67%)	2.74 (1.42; 5.35)
**Sterilization**				<0.001
No	807 (82.10%)	506 (75.75%)	301 (95.56%)	Ref
Yes	176 (17.90%)	162 (24.25%)	14 (4.44%)	0.15 (0.08; 0.25)
**Habitat**				<0.001
Garden house/farm	96 (9.74%)	85 (12.67%)	11 (3.49%)	Ref
Kennel	603 (61.16%)	337 (50.22%)	266 (84.44%)	6.01 (3.27; 12.2)
Apartment	254 (25.76%)	222 (33.08%)	32 (10.16%)	1.10 (0.54; 2.40)
Rehale	33 (3.35%)	27 (4.02%)	6 (1.90%)	1.73 (0.54; 5.07)
**Size**				<0.001
Small	121 (12.29%)	97 (14.48%)	24 (7.65%)	Ref
Medium	346 (35.16%)	171 (25.52%)	175 (55.73%)	4.11 (2.54; 6.87)
Large	517 (52.54%)	402 (60.00%)	115 (33.63%)	1.15 (0.71; 1.92)
**Last vaccine**				<0.05
Monovalent	667 (93.29%)	559 (92.40%)	108 (98.18%)	Ref
Polyvalent	48 (6.71%)	46 (7.60%)	2 (1.82%)	0.24 (0.04; 0.80)
**Origin**				1.000
Eastern countries	10 (1.07%)	7 (1.05%)	3 (1.12%)	Ref
Rest of Europe and America	923 (98.93%)	657 (98.95%)	266 (98.88%)	0.92 (0.25; 4.53)
**State of health**				0.017
Not optimal	118 (12.00%)	92 (13.77%)	26 (8.25%)	Ref
Optimal	865 (88.00%)	576 (86.23%)	289 (91.75%)	1.77 (1.13; 2.85)

**Table 2:** Relationship between the seroconversion level in IU/mL (considering 4 categories) and the variables studied.

Variable	Number of Animals N = 1060	Number of Animals with Titers < 0.125 N = 209	Number of Animals with Titers < 0.5 N = 143	Number of Animals with Titers ≥ 0.5 N = 390	Number of Animals with Titers > 4 N = 318	*p. Trend*
**Population (Main group)**						0.000
Shelters	269 (25.38%)	132 (63.16%)	73 (51.05%)	48 (12.31%)	16 (5.03%)	
Vet clinics	383 (36.13%)	27 (12.92%)	22 (15.38%)	152 (38.97%)	182 (57.23%)	
Animal nursery	60 (5.66%)	18 (8.61%)	14 (9.79%)	24 (6.15%)	4 (1.26%)	
Research facilities	28 (2.64%)	7 (3.35%)	6 (4.29%)	15 (3.85%)	0 (0.00%)	
Working	320 (30.19%)	25 (11.96%)	28 (19.58%)	151 (38.72%)	116 (36.48%)	
**Behavior**						<0.001
Not sociable	112 (11.36%)	14 (7.49%)	6 (4.68%)	36 (10.02%)	56 (18.01%)	
Sociable	873 (88.63%)	173 (92.51%)	122 (95.31%)	323 (89.97%)	255 (81.99%)	
**Dangerousness**						0.007
No	947 (96.14%)	175 (93.58%)	119 (92.97%)	350 (97.49%)	303 (97.43%)	
Yes	38 (3.86%)	12 (6.42%)	9 (7.03%)	9 (2.51%)	8 (2.57%)	
**Sterilization**						0.000
No	807 (82.10%)	179 (95.72%)	122 (95.31%)	294 (82.35%)	212 (68.17%)	
Yes	176 (17.90%)	8 (4.28%)	6 (4.69%)	63 (17.65%)	99 (31.83%)	
**Habitat**						<0.001
Garden house/farm	96 (9.74%)	5 (2.67%)	6 (4.69%)	42 (11.67%)	43 (13.83%)	
Kennel	603 (61.16%)	160 (85.56%)	106 (82.81%)	208 (57.78%)	129 (41.48%)	
Apartment	254 (25.76%)	21 (11.23%)	1 (8.59%)1	89 (24.72%)	133 (42.77%)	
Rehale	33 (3.35%)	1 (0.53%)	5 (3.91%)	21 (5.83%)	6 (1.93%)	
**Size**						0.033
Small	121 (12.29%)	15 (8.02%)	9 (7.08%)	34 (9,47%)	63 (20.26%)	
Medium	346 (35.16%)	113 (60.43%)	62(48.82%)	95 (26.46%)	76 (24.44%)	
Large	517 (52.54%)	59 (31.55%)	56 (44.09%)	230 (64.07%)	172 (55.3%)	
**Last vaccine**						0.005
Monovalent	667 (93.29%)	53 (96.36%)	55 (100.00%)	293 (94.52%)	266 (90.17%)	
Polyvalent	48 (6.71%)	2 (3.64%)	0 (0.00%)	17 (5.48%)	29 (9.83%)	
**Origin**						0.819
Eastern countries	10 (1.07%)	3 (1.94%)	0 (0.00%)	3 (0.84%)	4 (1.30%)	
Europe and America	923 (98.93%)	152 (98.06%)	114 (100.00%)	353 (99.16%)	304 (98.70%)	
**State of health**						0.003
Not optimal	118 (12.00%)	12 (6.41%)	14 (10.93%)	45 (12.53%)	47(15.27%)	
Optimal	865 (88.00%)	175 (93.58%)	114 (89.06%)	314 (87.47%)	262 (84.79%)	

## Data Availability

The data presented in this study are available upon request from the corresponding author.
